# Patient engagement strategies in digital health interventions for cancer survivors: A scoping review

**DOI:** 10.1371/journal.pdig.0000871

**Published:** 2025-05-30

**Authors:** Maria Ren, Camila E. Orsso, Homa Ghomashchi, Bruna R. da Silva, Christa Aubrey, Ingrid Nielssen, Sophia Pin, Margaret L. McNeely, Puneeta Tandon, Carla M. Prado

**Affiliations:** 1 Department of Agricultural, Food & Nutritional Science, University of Alberta, Edmonton, Canada; 2 Department of Obstetrics & Gynecology, University of Alberta, Edmonton, Canada; 3 Division of Gynecologic Oncology, Cross Cancer Institute, Edmonton, Canada; 4 Department of Community Health Sciences, University of Calgary, Calgary, Canada; 5 Department of Physical Therapy/Department of Oncology, University of Alberta, Edmonton, Canada; 6 Department of Medicine, University of Alberta, Edmonton, Canada; The University of Hong Kong, HONG KONG

## Abstract

Individuals can face various mental and physical health challenges after a cancer diagnosis. Digital health platforms can address some of these challenges by providing self-management tools for improving lifestyle behaviors, while reducing the burden on healthcare systems and enhancing healthcare access to underserved populations. Involving individuals with a history of cancer, termed here as “cancer survivors”, in the development and evaluation of digital health platforms can improve their effectiveness. This scoping review aimed to explore the state of patient engagement in research on digital health platforms for cancer survivors, including strategies for engagement, characteristics, and identifying gaps and barriers. A systematic search was conducted in OVID Medline, OVID EMBASE, and Scopus from inception until May 2023. The review followed Joanna Briggs Institute’s guidance for scoping reviews. Eligible studies actively involved cancer survivors in the development or evaluation of digital health platforms. These studies focused on self-management digital health platforms delivering nutrition, physical activity, and/or mental health interventions. Reporting of patient engagement was evaluated according to the Guidance for Reporting Involvement of Patients and the Public 2 (GRIPP2). The search strategy captured 7 studies using various patient engagement approaches, with patient and public involvement being the most frequently used (43%, n = 3). Studies were conducted in 6 countries and most focused on the development or evaluation of web-based digital health platforms (71%, n = 5). Few studies reported all elements of GRIPP2’s reporting checklist (29%, n = 2). We further identified barriers and areas of improvement for patient engagement in digital health research. Patient engagement improves digital health platforms, but few studies have meaningfully included patients, therefore reporting and evaluation of patient engagement is necessary to support its adoption in digital health research projects. In addition to exploring the gaps in patient engagement practices, this scoping review serves as a foundation for future research to advance patient-oriented digital health interventions for cancer survivors.

## Author summary

Cancer survivors often face challenges related to their mental and physical wellbeing, including higher risks of diseases such as cardiovascular disease and obesity. Despite these risks, they frequently lack support from healthcare professionals regarding healthy eating, staying active, and mindfulness. Digital health platforms can assist cancer survivors in overcoming these challenges by providing tools for self-managing a more optimal, healthy lifestyle. The perspectives of cancer survivors and their caregivers are crucial for improving digital health platforms, particularly when they actively participate in research teams. With this in mind, we conducted a review to examine how digital health platform research has included cancer survivors on research teams and to identify ways to enhance their involvement. Despite the benefits of including cancer survivors on research teams, we found that few studies have involved them. Additionally, there is limited reporting on the methods used to engage cancer survivors in research. This study highlights the importance of effectively reporting and evaluating patient engagement to enhance overall patient involvement. Our review assessed the current state of patient engagement in digital health platform research and provided insights to guide future research teams in involving cancer survivors more effectively.

## Introduction

Cancer survival rates in Canada and other high-income countries have substantially improved in the past three decades [1–3]. However, both individuals undergoing cancer treatment and those in clinical remission, commonly referred to as cancer survivors [4], frequently encounter numerous challenges. Mental and physical health effects related to cancer and its treatments are persistent, impacting cancer survivors over a long-term [5].

Cancer and its treatments are associated with a wide range of long-term health complications. These may include fatigue, mental health challenges such as anxiety or depression, cognitive impairment, weight gain or muscle loss, and sleep disturbances [5,6]. Some cancer types and treatments also increase the risk of cardiovascular disease (CVD), which has been linked to elevated mortality in cancer survivors [7–10]. Given the varied health risk, targeted and effective lifestyle interventions are essential to support long term well-being.

Lifestyle modifications, including physical activity, nutritional strategies and mental health support, play a crucial role in managing many of these complications. These interventions can improve quality of life, reduce psychological distress and help mitigate risks of chronic conditions, such as CVD [11–14]. For example, regular physical activity has been shown to improve cardiovascular health, reduce cancer-related fatigue and lower all-cause and cancer-specific mortality [15,16]. Nutritional strategies further support these benefits and are considered one of the primary methods for preventing chronic diseases [17–19]. Notably, higher diet quality at diagnosis has been associated with a lower risk of CVD events [20]. Furthermore, mental health is a critical component of overall well-being for cancer survivors.

Addressing mental health can enhance adherence to physical activity and dietary interventions, thereby amplifying their positive effects on preventing comorbid conditions and overall quality of life [21,22]. However, providing these comprehensive interventions—which include physical, nutritional, and mental health support—is crucial for high-quality survivorship care but can be costly and dependent on the availability of healthcare professionals [23]. This underscores the need for programs capable of delivering multimodal, person-centered, and effective healthcare to a broad patient base at a lower cost.

Digital health platforms have the potential to alleviate the burden on healthcare systems and help overcome barriers to healthcare access in underserved populations [24]. Improvements in physical activity have been reported in cancer survivors that use digital health interventions [25]. However, more evidence is needed to assess the effectiveness of digital interventions for nutrition and mental health, particularly in conjunction with physical activity [26]. Regardless of the type of intervention delivered, digital health platforms are most effective if they are tailored to the individual or population group in terms of design and relevance [27]. Involving individuals who have lived experience of chronic conditions or their informal caregivers (collectively termed ‘patients’ [28]) in the development and evaluation of these platforms can meaningfully improve their overall impact.

Including patients in the research process can yield many benefits, such as increasing the relevance of interventions to specific groups and supporting participant retention, which are crucial advantages for digital health programs [29]. While web-based digital health interventions have demonstrated more immediate effectiveness than offline interventions, high dropout rates diminish their long-term success [30]. By incorporating patient insights and perspectives from the beginning and throughout the development and evaluation phases, digital health interventions can better meet patient needs, enhancing their effectiveness in delivering high-quality healthcare.

The Canadian Institutes of Health Research (CIHR) has emphasized the importance of involving patients in research, leading to the development of the Strategy for Patient-Oriented Research (SPOR) [28]. This strategy highlights patient engagement as a “meaningful” and “active” partnership between researchers and patients, where patients contribute their insights and lived experience throughout the research process. Similarly, health research institutions worldwide have initiated programs advocating for the integration of patients in research teams [31].

This scoping review focused on examining patient engagement in digital health platforms for lifestyle behavior change for cancer survivors. Our goals were to: 1) identify strategies used to engage patients as partners in research; 2) determine what makes patient engagement effective; and 3) explore challenges and gaps in involving patient partners in research. Throughout this review, we used CIHR’s definitions for terms such as patient engagement, patient partner, and patient, **Table 1** [28]. By adopting CIHR’s broad definitions, we aim to cover a wide range of approaches and terminologies related to patient roles in research.

**Table 1:** Canadian Institutes of Health Research (CIHR) strategy for patient-oriented research definitions and patient partners roles [28].

	Description
**Terminology**	
**Patient-oriented research**	“Patient-oriented research refers to a continuum of research that engages patients as partners, focusses on patient-identified priorities and improves patient outcomes. This research, conducted by multidisciplinary teams in partnership with relevant stakeholders, aims to apply the knowledge generated to improve healthcare systems and practices.” [28]
**Patient engagement**	“Meaningful and active collaboration in governance, priority setting, conducting research and knowledge translation.” [28] I.e. Moving patients beyond passive participants to active partners in research.
**Patient**	“…individuals with personal experience of a health issue and informal caregivers, including family and friends.” [28] In patient-oriented research this can also include the collective voice of specific affected communities.
**Patient partner**	Patients who are actively involved in the research process as collaborators.
**Patient partner roles**	
**Research committee member**	“Plan, design, and guide a project as it progresses.” [28]
**Competent patient engagement researchers**	“Patients with mastery of research skills engage other patients and incorporate their ideas into research.” [28]
**Contributors**	“Contribute to the different steps of the research process or lifecycle.” [28]
**Supporters of participant-friendly research studies**	“Improve access to patients and the reach of recruitment.”[28]

## Methods

This scoping review was conducted following a pre-established protocol, adhering to the methodological framework for scoping reviews proposed by the Joanna Briggs Institute (JBI) [32]. This framework was refined from a previous one developed by Arksey and O’Malley [33]. The final protocol is available in the supplementary material (**S1** Protocol). Reporting of the review conformed to the Preferred Reporting Items for Systematic Reviews and Meta-Analyses extension for Scoping Reviews checklist, (PRISMA-ScR; S1 PRISMA Checklist) [34] aligning with the JBI methodological framework. The JBI guidance for scoping reviews recommends the use of the PRISMA checklist and flowchart to ensure rigorous and transparent reporting. While both JBI and PRISMA emphasize systematic and comprehensive reporting, the PRISMA-ScR extension is specifically designed to accommodate the broader objectives of scoping reviews, which often include mapping the evidence, identifying gaps, and providing an overview of the existing literature, rather than focusing solely on the synthesis of findings. To ensure methodological rigor and clarity in our scoping review, we adhere to both JBI and PRISMA-ScR guidelines. This approach aligns our review with best practices for transparency and reproducibility.

### Research questions

This scoping review was guided by a central question: Has patient engagement been incorporated into research on digital healthcare interventions for managing nutrition, physical activity, and/or mental health in cancer survivors? To further explore this inquiry, the review sought to answer a series of questions, facilitating the literature review and data extraction processes: (1) What strategies have been used to engage cancer survivors, or their caregivers, as active partners in digital health platform research? (2) How effective have these strategies been in facilitating patient engagement? And, what are the characteristics of successful patient engagement in digital health interventions? (3) What are some challenges in engaging cancer survivors or their caregivers as patient partners in digital health platform research? Which aspects of patient engagement research in digital health platform research need further exploration?

### Eligibility criteria

The Population, Concept, and Context (PCC) framework was used to formulate the eligibility criteria (**Table 2**). Selected articles involved cancer survivors, or their caregivers, actively as patient partners in studies that focused on developing digital health platforms or evaluating already existing ones. Original articles and study protocols were included. We excluded thesis, case reports, case series, narrative reviews, systematic reviews, meta-analysis, conference abstracts, and non-peer-reviewed articles, as these sources of evidence would not identify specific characteristics of patient engagement. The search was limited to the English language.

**Table 2:** Eligibility criteria following the Population, Concept, Context (PCC) framework.

Variable	Description
**Population**	Studies included adult survivors of cancer as the user demographic of digital health platforms. Classification of cancer survivorship was broad, encompassing patients at all stages of disease after their initial diagnosis, according to the National Cancer Institution’s definition of cancer survivors [4]. The search excluded all research involving infants, children, and adolescents.
**Concept**	Research considered to have used patient engagement strategies that actively engaged patients or their informal caregivers at any step of the research process, from informing prospective digital health platforms, to development, validation, and knowledge translation. The inclusion of patient partners was explicitly outlined and aligned with one or more of the roles described in **Table 1**. At the stage(s) where patient partners were engaged, they could not have been part of the data set, except in evaluation of patient engagement.
**Context**	The studies developed or evaluated digital health platforms delivering healthcare interventions in the following areas, independently or in combination: nutrition, physical activity, and mental health. Digital interventions used web-based platforms (i.e., functioned within a web browser) or mobile applications (i.e., operated within a phone app). Wearable technology, health record tools, and text messaging services were only considered if they were used in conjunction with a web-based platform or a mobile application.

### Search strategy

The search strategy began with a preliminary search on Ovid MEDLINE to identify relevant articles and derive index terms. Secondly, a refined search strategy was formed; first for Ovid MEDLINE, then adapted for Ovid EMBASE and Scopus. The adapted search strategy for each database was made through modification of the established keywords and index terms. Search terms related to the concepts of ‘patient engagement’, ‘digital health’, ‘lifestyle (i.e., nutrition, exercise, and mental health) intervention’, and ‘cancer’ formed the basis of the search strategy. The search was conducted from inception (earliest available records) to May 23, 2023. S1 Table outlines the search strategy used.

### Study selection

The search results were uploaded into Covidence online software (Veritas Health Innovation Ltd) for study selection. Duplicate articles were automatically removed before screening. A standard screening procedure with the eligibility criteria (**Table 2**) was established before the screening process began. Three reviewers independently screened, in duplicate, the titles and abstracts followed by the full texts of eligible articles. Given the lack of clarity in the title and abstract alone, eligibility of patient engagement and healthcare intervention were primarily identified through full-text screening. In the case of conflicts, reviewers re-screened articles and discussed the eligibility; disagreements were either solved by consensus or by a third reviewer.

### Data extraction

The data extraction form was adapted from a template provided by the Joanna Briggs Institute to align with our objectives [32]. Prior to the main data extraction phase, we conducted a pilot test on the form using two articles. The test was conducted by one author and reviewed by another to ensure the form’s effectiveness in capturing necessary data to achieve our objectives. Following this, the form was uploaded to Covidence, and the data extraction process was completed by two independent reviewers using the platform, with any discrepancies resolved through consensus. Data extracted from selected studies included: citation details, title, country, type of study, condition of participants, age of participants, number of research participants, number of patient partners, type of digital intervention, area(s) of health addressed by the intervention, patient engagement strategies used, stages of the CIHR SPOR research lifecycle involved [35,36], and the components of the Guidance for Reporting Involvement of Patients and the Public 2 (GRIPP2) short form checklist [37].

### Data analysis

Statistical and thematic analysis, as well as appraisal of methodological quality did not take place because the aim of this review was exploratory. Study characteristics were synthesized in tabular and descriptive formats. Research lifecycle stages were interpreted based on the CIHR’s description of patient roles in each stage, synthesized, and tabulated [35,36]. The GRIPP2 short form checklist was used to assess the reporting of patient engagement in studies [37]. Developed to provide a standardized approach, GRIPP2 enables researchers to consistently document the involvement of patients and the public [37], ensuring that these contributions are clearly recorded. In our study, we summarized and tabulated data from the checklist’s components, allowing us to descriptively synthesize the strategies, results, and barriers to patient engagement. Additionally, patient engagement characteristics and terminology were also tabulated and described.

## Results

### Study selection

**Fig 1:**
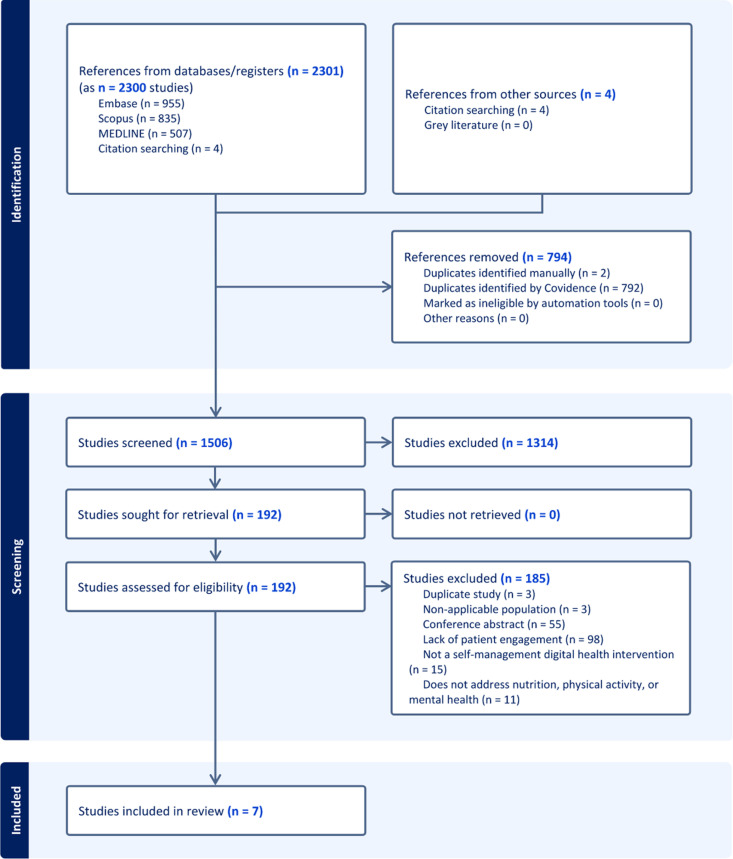
PRISMA study selection flow chart.

The initial search resulted in 2,301 articles (**Fig 1**). Following title and abstract screening, 192 studies were selected for full-text review, and 7 articles met the eligibility criteria [38–44]. Upon full-text review, 98 articles were excluded for not incorporating or describing patient engagement. While many used patient engagement terminologies, they did not involve patients as active partners in the research. Exclusion of remaining articles was due to various reasons: conference abstracts (55 articles); lack of a digital health platform with self-management components (15 articles); failure to address nutrition, physical activity, or mental health of cancer survivors (11 articles); manual duplicates (3 articles); studies not focusing on cancer survivorship (3 articles).

### Summary of study characteristics

**Table 3** summarizes the characteristics of included studies published between 2015 and 2023. Two studies were conducted in the United States [38,42] and the other countries included Australia [44], Canada [40], Germany [43], the Netherlands [41], and the United Kingdom [39], with 1 study each. Most articles were study protocols, either for randomized controlled trials [41,43,44] or a mixed-methods study [40]. A randomized pilot trial [42], as well as 2 formative studies [38,39] were included. The formative studies employed a descriptive approach to detail how patients were involved at various stages of digital health platform development. The funding sources for these studies included the National Institutes of Health [38,42], Dutch Cancer Society [41], Yorkshire Cancer Research [39], Victorian Cancer Agency [44], Federal Ministry of Education and Research [43], and the Canadian Cancer Research Alliance [40].

**Table 3:** Characteristics of studies involving patients in research on digital health platforms for lifestyle behavior change among cancer survivors.

First author year	Country	Intended users of the digital health platform	Type of Digital Health Platform	Study Aims	Health Intervention	Study type	Study Funding
**VandeWal 2015** [41]	Netherlands	Breast, colorectal, and prostate cancer survivors	Web-based blended with face-to-face	Evaluate the efficacy of cognitive behaviour therapy in reducing fear of cancer recurrence	Cognitive behavioral therapy related information materials, assignments, and assessments to modify thought patterns and dysfunctional behaviors	Protocol for a randomized controlled trial	Dutch Cancer Society
**VanBlarigan 2020** [42]	United States	Colorectal cancer survivors	Web-based with text-messaging	Evaluate the feasibility and acceptability of a remotely delivered dietary intervention using a web-based platform and text messaging	Nutrition recommendations, goal setting, meal planning Dietary intake tracking Text-message reminders	Randomized pilot trial	National Institutes of Health (NIH)
**Adler 2022** [38]	United States	Any cancer survivors living with disabilities	Mobile application	Design a mobile health application for cancer survivors to improve their quality of life and increase their self-efficacy to manage cancer as a chronic condition	Mindfulness and self-management of health e-modules	Formative, co-design study	National Institutes of Health (NIH)
**Curry 2022** [39]	United Kingdom	Lung cancer survivors	Web-based	Describe how patients were involved in the co-design process of adapting an existing exercise website to meet lung cancer survivors’ needs	Personalized physical activity programming and education Guided behavior change techniques to overcome barriers to physical activity	Formative, commentary	Yorkshire Cancer Research
**Hanna 2018** [44]	Australia	Upper-gastrointestinal tract (oesophagus, stomach, and pancreas) cancer survivors	Mobile application	Provide intensive dietetic intervention using telephone or mobile health application delivery close to the time of diagnosis for upper gastrointestinal cancer and assess the effect on quality-adjusted life years.	Track weight, nutrition impact symptoms, and dietary intake Reminders to promote behavior change and adherence	Protocol for a randomized controlled trial	Victorian Cancer Agency
**Heinen 2022** [43]	Germany	Any cancer survivors	Web-based	Develop a web-based psycho-oncological intervention to equip patients with skills to manage cancer-related challenges	Self-evaluation of distress level, skills, and mindfulness Track mental health progress Psychoeducational e-modules with videos, skills training, and audio-guided mindfulness exercises Mindfulness plans and motivational messages	Protocol for a randomized controlled trial	Federal Ministry of Education and Research
**Bernard 2023** [40]	Canada	Gynecological (uterine, cervical, vulvar, and vaginal) cancer survivors	Web-based	Identify preferences, barriers and facilitators from patients with gynaecological cancer regarding virtual pelvic healthcare survivorship care	Physical activity e-module with pelvic floor muscle training	Protocol for a mixed-methods qualitative study	Canadian Cancer Research Alliance

Web-based health platforms were the focus of 5 studies [39–43]. The remaining 2 studies developed or evaluated mobile applications [38,44]. Two studies combined mixed modes of healthcare delivery, employing face-to-face [41] or text-messaging [42] services alongside a web-based platform. All studies focused primarily on the delivery of a single aspect of health, including mental health in 3 studies [38,41,43], nutrition in 2 studies, [42,44] and physical activity in 2 other studies [39,40]. The types of interventions used within the digital health platforms were diverse (**Table 3**).

### Lived experiences

Studies actively involved patients with experience of different cancer types in a single (3 studies) [40,42,44] or various (3 studies) [38,41,43] anatomical locations, or of one specific cancer type (1 study) [39]. Across all studies, patient partners’ lived experiences mirrored those of the digital health platforms investigated. Only one study included informal caregivers alongside individuals who had experienced cancer [39]. Two studies provided details on the characteristics of patient partners beyond cancer [38,43]. Both studies noted a predominance of female over male cancer survivors. Heinen et al. reported that all patient partners were already involved in cancer support groups before joining the study [43]. Patient partners in Adler et al. were active in cancer and disability advocacy organizations [38]. They also characterized the patient partners as individuals living with long-term physical, cognitive, or social effects of cancer, who were cancer-free, and completed primary treatment in at least the last 5 years. Additionally, this study detailed the varied professional backgrounds of the patient partners, including social work, graphic design, research support, and rehabilitation medicine.

### Patient engagement terminologies and strategies

A range of patient engagement approaches and frameworks were identified, with varied terminology used to describe patients and patient engagement methods (**Table 4**). Patient and Public Involvement (PPI) emerged as the most identified approach [39,40,43]. Among studies adopting a PPI approach, one study referenced the British National Institute for Health Research’s *Involve* guidelines as their guiding framework [43]. Another study utilized terminology for two approaches: Patient-Oriented Research and PPI [40]. Additional patient engagement approaches noted were Human-Centered Design Methodology [42], Citizen Science [38], and Community Involvement [44]. One study did not specify any approach [41].

**Table 4:** Terminology and definitions of patient engagement approaches used in studies on digital health platforms for lifestyle behavior change among cancer survivors.

First author year	Patient engagement approach	Framework	Terms used to describe patient partners	Patients engaged	Number of patients engaged
**VandeWal 2015** [41]	Not specified	Not specified	Patient representatives; advisory committee member	Cancer survivors	3
**VanBlarigan 2020** [42]	Human-centered design methodology	Not specified	Patient advisory board member	Cancer survivors	5
**Adler 2022** [38]	Citizen scientist approach	Not specified	Survivor scientists	Cancer survivors	5
**Curry 2022** [39]	Patient and Public Involvement	Not specified	PPI members	Cancer survivors, caregivers, and family members	Not specified
**Hanna 2018** [44]	Community involvement	Not specified	Consumer representatives; advisory group member; advisory and safety committee member; project advisory committee member	Cancer survivors	Not specified
**Heinen 2022** [43]	Patient and Public Involvement	British National Institute for Health Research: *Involve* guidelines [45]	Patient advisory council member	Cancer survivors	5
**Bernard 2023** [40]	Patient and Public Involvement; Patient-Oriented Research	Canada Institutes of Health Research: Strategy for Patient Oriented Research – Patient Engagement Framework [28]	Patient advisors	Cancer survivors	Not specified

Regarding patient engagement, one study [41] involved 3 patients as partners, while 4 studies involved 5 patients each as partners [38,41–43]. Two studies did not disclose the number of patient partners involved [39,40,44]. Terminology to describe patient partners varied: 1 study each called them patient representatives [41], others referred to them as survivor scientists [38], consumer representatives [44], patient advisors [40], and PPI members [39]. In 4 studies, patients were involved in advisory groups [41–44]. Two studies featured patient involvement in working groups alongside healthcare professionals [41,44]; with 1 of these studies also including research staff in the working group [44].

### Patient engagement in the research lifecycle

**Fig 2:**
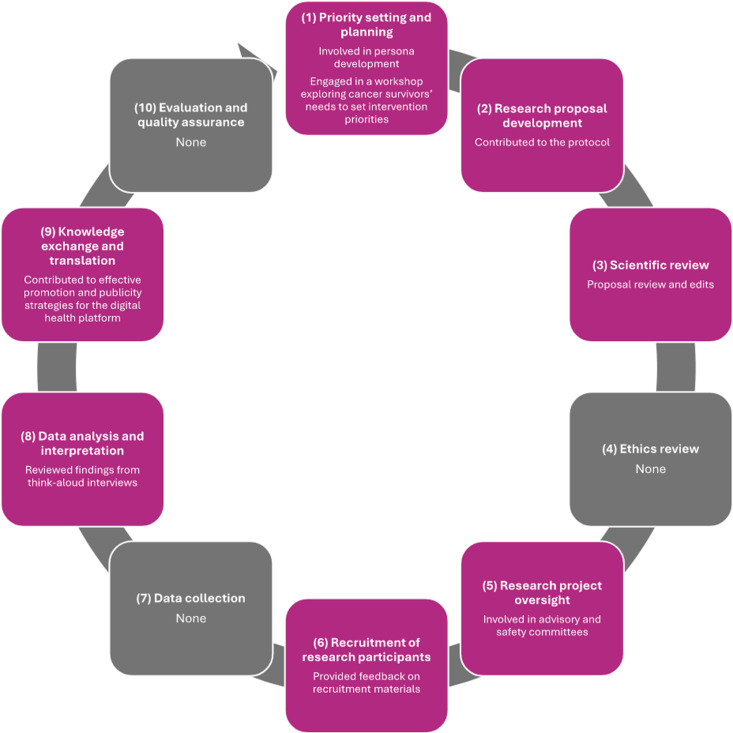
Examples of patient engagement activities across research lifecycle stages. (1) Patient partners were involved in persona development [38] and engaged in a workshop exploring cancer survivors’ needs to set intervention priorities [39]. (2) Patient partners contributed to the research protocol [40] and (3) reviewed a research proposal and made edits [40]. (4) No activities were reported within this research lifecycle stage. (5) Patient partners were involved in advisory and safety committees [44]. (6) Patient partners provided feedback on recruitment materials [40]. (7) No activities were reported within this research lifecycle stage. (8) Patient partners reviewed findings from think-aloud interviews [39]. (9) Patient partners contributed to effective promotion and publicity strategies for the digital health platform [43]. (10) No activities were reported within this research lifecycle stage. Research lifestyle activity examples derived from: Canadian Institutes of Health Research. Ethics Guidance for Developing Partnerships with Patients and Researchers [Internet]. 2020 [cited 2023 Dec 15]. Available from: https://cihr-irsc.gc.ca/e/51910.html. Research lifecycle derived from: Anderson JA. Research Ethics Broadly Writ: Beyond REB Review. Health Law Rev. 2011;19.

Patients were involved in various stages of the research lifecycle (**Table 5**) [35,36]. Five studies focused on patient engagement primarily in the development of a digital health platform [38,39,41–43]. Patient partners were included in only 1 stage of the research lifecycle in 2 studies [38,41], while 5 studies engaged patient partners across 2 or more stages [39,40,42–44]. The stage most frequently featuring patient engagement was ‘development of the research proposal’ occurring in 5 studies [40–44]. This was followed by ‘knowledge exchange and translation’ in 4 studies [39,40,43,44], ‘oversight of a research project’ in 3 studies [42–44], and ‘data analysis and interpretation’ also in 3 studies [39,40,44]. Notably, the stages: ‘ethics review’, ‘data collection’, and ‘evaluation and quality assurance’ saw no patient engagement. Fig 2 (S2 Table) provides illustrative examples of patient engagement characterized at each research lifecycle stage.

**Table 5:** Patient engagement at each stage of the research lifecycle in studies on digital health platforms for lifestyle behavior change among cancer survivors.

First author year	VandeWal 2015 [41]	VanBlarigan 2020 [42]	Adler 2022 [38]	Curry 2022 [39]	Hanna 2018 [44]	Heinen 2022 [43]	Bernard 2023 [40]
**Priority setting and planning**			X	X			
**Development of research proposal**	X	X			X	X	X
**Scientific review**							X
**Ethics review**							
**Oversight of a research project**		X			X	X	
**Recruitment of research participants**					X		X
**Data collection**							
**Data analysis and interpretation**				X	X		X
**Knowledge exchange and translation**				X	X	X	X
**Evaluation and quality assurance**							

### Reporting of patient engagement using GRIPP2

Reporting of the GRIPP2 short form checklist elements by the studies is detailed below and in **Table 6**. Only 2 studies reported all elements of the checklist [38,39].

**Table 6:** Components of the Guidance for Reporting Involvement of Patients and the Public 2 (GRIPP2) short form reported by studies involving patients in research on digital health platforms for lifestyle behavior change among cancer survivors.

First author year	Aim	Methods	Study results	Discussion and conclusions	Reflections/ critical perspective
**VandeWal 2015** [41]	Not reported	3 cancer survivors were recruited to form an advisory committee Provided feedback by reviewing therapy content Proposed comments, ideas, and suggestions to improve the intervention Completed a questionnaire to evaluate the website Engaged in usability testing	The content was revised based on the advisory committee’s feedback	Not reported	Not reported
**VanBlarigan 2020** [42]	Not reported	5 colorectal cancer survivors were recruited to form a patient advisory board Engaged in intervention development and tested a prototype Participated in semi-structured interviews about the intervention	The intervention was revised based on the results of the semi-structured interview	Not reported	Not reported
**Adler 2022** [38]	To ensure that the platform and design meet the needs and preferences of the target end users	5 cancer survivors with known disabilities collaborated with the research and development team Engaged in 3 co-design workshops: (1) persona development, (2) prototype ideation, and (3) prototype development Workshops took place using Zoom Web-based tools were used including Zoom breakout rooms, poll and chat features, Slido, word clouds, and Miro Cancer survivors were assigned tasks to work with the research team based on their interests	The cancer survivors helped identify challenges faced by cancer survivors with disabilities and considerations for designing the mobile health application	Working with the cancer survivors gave the research team a deeper level of empathy and better understanding of the target users	Reflections were made on creating an inclusive environment, making the workshops engaging, and timeliness
**Curry 2022** [39]	To ensure that the digital health intervention and new content was appropriate for lung cancer survivors	Cancer survivors were recruited through online recruitment flyers and an existing Patient and Public Involvement (PPI) network 3 workshops took place to review and develop content for the platform, and included 4 areas: (1) understanding of physical activity concerning lung cancer, (2) barriers to engaging in physical activity and digital technology, (3) creating clear materials for study participants, (4) module development within Exercise Guide UK Cancer survivors reviewed findings from Think-Allowed interviews conducted with other lung cancer survivor participants Patient engagement sessions took place using Zoom 2 cancer survivors were co-authors of the current article	Cancer survivors reported their involvement to be valuable for themselves The final prototype of the platform was more appropriate, had greater clarity, and overall usability for lung cancer survivors	Working with the cancer survivors helped address barriers about physical activity and digital technology Doctoral students can benefit from patient input Cancer survivors can benefit from their involvement Working with the cancer survivors was beneficial for intervention success	Cancer survivors reported that their involvement positively impacted their perceptions of lung cancer and improved their understanding of PPI in health research The doctoral student leading the project noted that setting up a PPI group earlier on in the process would likely have been beneficial Virtual PPI may have excluded some individuals Receiving criticism was difficult for the doctoral student
**Hanna 2018** [44]	Not reported	Cancer survivors took part in an advisory group, as well as the advisory and safety committee The advisory group gave feedback on recruitment procedures and the intervention The advisory and safety committee was proposed to meet twice a year to review the progress of the trial and discussed any unforeseen or adverse events; they were also proposed to review trial results at the end of the study, help interpret findings, and communicate results	Feedback from the advisory group meetings was implemented in the final protocol	Not reported	Not reported
**Heinen 2022** [43]	Advise the project team in planning and optimization of the intervention and associated studies	5 patients who were members of cancer support groups were contacted by the psycho-oncological units of university hospitals and formed a patient council The patient council took part in regular meetings and communication with the study team Methods of communication included online meetings, phone calls, and e-mail exchange The patient council advised the research team on optimization of the digital training and study process, creating a patient-friendly user experience, and dissemination of the project among patients and self-help groups The patient council was regularly asked for feedback after intervention updates	Feedback from cancer survivors helped improve the intervention Continuous feedback from the cancer survivors helped identify user difficulties, constructive solutions, and improved dissemination of the intervention to cancer-affected individuals	Not reported	Not reported
**Bernard 2023** [40]	Adapt research methods and means used to represent and disseminate results to patients fairly and effectively	Cancer survivors were actively involved in research activities according to their preferred level of engagement Cancer survivors were involved in identifying important research questions, reviewed the protocol and provided comments and suggests, and reviewed survey questions and recruitment materials Cancer survivors will be involved in further research stages, such as data analysis and reviewing themes	Not reported	Not reported	Not reported

### Aims of patient engagement

Four studies clearly stated the aims of patient engagement within the research project. In 2 studies, the objective was to ensure the digital health platforms met end-user needs [38,39]. Additional aims included providing advice to the research team [43] and ensuring equitable dissemination of results [40].

### Patient engagement methods

#### Recruitment of cancer survivors.

Three studies described the recruitment of patient partners. In 1 study, cancer survivors were invited via existing connections of the research team [38]. In Heinen et al., patients from cancer support groups were contacted by the psycho-oncological units of participating university hospitals [43]; and additional patient partners were nominated by a cancer self-help organization. Another study distributed a recruitment flyer on social media and ‘*Involve Hull*’, a PPI network, to reach survivors [39].

#### Research activities.

All studies described the patient partners’ research activities. Two studies had patient partners test the digital health platform or the intervention [41,42]. One study engaged patient partners in semi-structured interviews not used for publication [42], and another involved patient partners in questionnaire and usability testing through a think-aloud procedure without publishing the data [41]. Patient partners in another study reviewed findings from think-aloud interviews conducted with research participants [39]. One study asked patient partners to choose their “level of engagement” [40]. Structured workshops in 2 studies facilitated activities like persona development [38], content generation for a mobile application [38], and prototype development for a digital health platform [38,39]. Patient partners were involved in regular study meetings [43], and advisor and safety committee meetings [44]. In Adler et al., patient partners also collaborated individually with researchers, contributing with content according to their expertise and interests [38].

#### Communication and meetings.

Virtual meetings were used in 3 studies for engaging with patient partners, with 2 specifying the use of Zoom [38,39]. One study employed additional web-based tools like Slido, word clouds, and Miro [38]. Another reported using phone calls and e-mail for communication [43]. The communication methods in 4 studies were not described [40–42,44].

### Results of patient engagement

Although no studies used a validated tool to evaluate patient engagement, 2 reported that patient partner feedback enhanced the digital health platform design [39,43] and dissemination [43]. Two studies found that patient partners highlighted challenges and solutions for cancer survivors [38,43], and in 1 study, they contributed to refining the study protocol [44]. Feedback from patient partners led to revisions in interventions in 2 studies [41,42]. Adler et al. reported detailed outcomes of workshops including persona development, and prototype ideation and development [38]. Curry et al. described specific platform revisions made during workshops with patient partners and noted that patient engagement positively impacted patient partners’ perceptions of their condition [39].

### Discussion and reflections on patient engagement

#### Facilitators for patient engagement.

Reflecting on patient engagement, 2 studies recognized that collaboration with patient partners enhanced researchers’ understanding of patient experiences [38,39]. In the study by Curry et al., patient partners felt that their partnership had a positive impact on their views of lung cancer, enriched by the shared experiences of others [39]. They also gained a deeper insight into PPI in health research. A doctoral student involved suggested that earlier involvement of patient partners could be more advantageous and recognized learning to accept criticism as part of the process. In Adler et al., efforts to create an inclusive environment were noted, including dedicating time for rapport-building, establishing ground rules, and assuring patients of their confidentiality [38].

#### Challenges and barriers for patient engagement.

During the Covid-19 pandemic, some studies faced challenges, with 2 reporting technology as an impediment to patient engagement [38,39]. Curry et al. observed that while virtual engagement could widen the scope of patient engagement and was found to be less intimidating by a patient partner, reliance on this method might alienate those lacking access to or skills in digital technology [39]. Conversely, virtual formats could also induce fatigue more readily, as identified by Adler et al. [38]. This study highlighted that engaging tools and features, such as Zoom’s breakout rooms, polling, and chat functions could maintain engagement during online workshops.

## Discussion

This scoping review is the first to investigate patient engagement in digital health platform research for cancer survivors. Patient engagement is becoming increasingly prevalent in research and is often a criterion in research grant proposals [46]. For example, patient engagement is a priority for the National Institutes of Health (NIH), which provides specific support services to encourage meaningful patient involvement in research [47]. Notably, the NIH funded two of the studies included in our review, highlighting its commitment to patient engagement research. When patient engagement is meaningfully incorporated in research, researchers can benefit in various ways, for example, by gaining new perspectives and a better understanding of research priorities and outcomes that matter to patients [48].

While the importance of patient engagement is widely recognized, our findings revealed that only a select number of studies meaningfully engaged patient partners. Patient partners were involved in different but few stages of the research lifecycle, predominantly in developing the research proposal. Only a few studies included patients in the early stages, such as priority setting and planning; however, one study acknowledged the potential advantages of earlier patient engagement [39]. The lack of early inclusion may suggest a gap in knowledge about how to meaningfully involve patient partners throughout the research process rather than a disregard for its importance [49]. Engagement strategies varied, with patients involved in regular meetings or specific research tasks like co-facilitating workshops, interviews, questionnaires, and testing digital health platforms. The Levels of Engagement are commonly used to measure the depth of patient engagement [50,51]. While we did not use this measure, many approaches described did not appear to be ‘partnerships’ or ‘deep inclusion’ [52]. For example, some studies primarily included patients in advisory groups or specific activities, suggesting a consultative rather than a partnership role [50,51]. Our findings aim to guide future research that seeks to involve patient partners in the development and evaluation of digital health platforms for cancer survivors.

Patient partners, drawing from their lived experiences, can refine research questions, select meaningful study outcomes, increase recruitment and retention rates, and facilitate the dissemination and implementation of study findings [29,53]. While the included studies did not explicitly mention any recruitment challenges, involving patient partners in the design process may have helped to address potential issues. Their contributions are essential for ensuring that the interventions are relevant and accessible to the target population. This approach could potentially lead to higher recruitment and retention rates compared to traditional research methodologies, where patient input is often limited. Our scoping review highlighted their key role in identifying challenges cancer survivors face, improving digital health platforms, and influencing study protocols. Nonetheless, there remains a need for more concrete evidence on the influence of patient engagement on society, the research community, and healthcare systems [54]. A noted deficiency in the literature is the evaluation of patient engagement through validated methods [49,55]—an observation consistent with our review. Despite this evaluation gap, incorporating patient partners’ perspectives intrinsically enhances research value, a fact supported by previous studies and reaffirmed by our review’s findings [56]. The growing practice of patient engagement requires more robust evaluation to support its continued adoption [57]. Due to the lack of evaluation, we were unable to achieve our objective of identifying characteristics of successful patient engagement, which underscores the repercussions of insufficient evaluation practices. To facilitate this evaluation, research leaders and patient partners have formulated The Learning Together Evaluation Framework for Patient and Public Engagement (PPE) in Research [58], offering researchers a flexible tool for planning and assessing patient engagement.

In comparison, an earlier systematic review explored meaningful patient and public engagement in digital health research, capturing all modes of digital health and various health conditions [59], not specifically targeting cancer survivors as our scoping review does. The review by Baines et al. encompassed a variety of patient-oriented strategies, where active patient involvement was not consistently implemented, with some studies treating patients solely as participants [59]. It addressed multiple health conditions, including CVD, mental disorders, and diverse age groups, illustrating that challenges in meaningful patient engagement extend beyond cancer and digital health [59]. Despite this comprehensive scope, the review similarly concluded that meaningful patient engagement remains underutilized in digital health research [59]. Consistent with our findings, Baines et al. observed that the absence of early patient involvement often presents barriers to effective engagement, underscoring the importance of co-design processes and thoughtful integration of digital tools to enhance patient engagement [59]. Moreover, Marzban et al. demonstrated that effective patient engagement across diverse populations—from general hospital patients to those with specific conditions like diabetes and high blood pressure—can significantly improve treatment outcomes and patient satisfaction [60]. These findings suggest that while challenges in patient engagement are widespread across various health domains, there are consistent opportunities and strategies that can be applied to enhance engagement and outcomes in digital health research.

Challenges and barriers to patient engagement identified during the Covid-19 pandemic were mainly due to the shift to exclusively virtual interactions. Challenges included patient partners’ lack of familiarity with the technology used for engagement and the shortened timeframe for thorough involvement [38], a finding corroborated by the previous systematic review [59]. Baines et al. identified additional barriers in digital health research, such as privacy and security concerns. The authors emphasized the importance of establishing trust and transparency to help reduce these concerns. They further suggested providing support for technology use and allocating sufficient time for patient partners to become acquainted with the digital health platforms. Both reviews also noted that the lack of digital access and literacy resulted in the exclusion of certain patient groups, potentially amplifying health disparities as digital health becomes increasingly mainstream (“digital divide”) [39,61]. Furthermore, cancer survivors may experience health and financial challenges [62] that can act as barriers to their involvement in research teams. Without providing appropriate supports to cancer survivor research partners, such as financial compensation and training opportunities [63], patients with lower socio-economic statuses may be excluded and patients may not be able to contribute meaningfully.

Studies included in this review used a variety of patient engagement approaches, but many did not describe specific frameworks. Research focusing on the co-development of a digital health platform may not follow typical health research cycle stages. As such, the suitability of Greenhalgh et al.’s recommendations to select and adapt frameworks according to the project context becomes evident [31]. They advocate for reviewing available frameworks and co-designing a framework with patient partners. Detailed descriptions of the chosen frameworks or the co-design process of implementing them can improve patient engagement in future digital health research. Without clear descriptions of the patient engagement framework employed, interpretations remain subjective and can lead to fragmented evidence on patient engagement, further complicating the evaluation of its effects [54].

This review found limited reporting of patient engagement that aligned with the GRIPP2 checklist, consistent with findings from broader studies on patient engagement [64]. Several included studies were protocols, which inherently limit our discussion. This is likely due to the ongoing nature and novelty of many digital health studies, which require detailed planning and protocol development. The GRIPP2 checklist can be used for both retrospective reporting and prospective planning of patient engagement [37]. Reports of patient engagement outcomes in our review were mainly authorial commentaries, lacking the foundation of standardized evaluation tools. Moreover, including patient partner perspectives is crucial for a full understanding of engagement outcomes [31,65]. Only one study included a patient partner commentary and co-authorship, suggesting that cancer survivors find personal value in being involved with research teams. The unique barriers of digital health research and the unique needs of patient partners with varied health conditions [66] require a clear and complete reporting of patient partner insights for enhancing engagement in digital health research involving cancer survivors [67].

Few studies reported the diverse characteristics of patient partners beyond their cancer experience, such as their cultural, age, geographical, and professional backgrounds. Recognizing patients’ unique identities is important to ensure a diverse range of perspectives, but researchers must also consider the challenges of providing such detailed information without compromising confidentiality. Given that research groups often involve small numbers of patient partners, and their names may be publicized as co-authors or acknowledgments, publishing a combination of identities and characteristics could inadvertently reveal excessive personal information. While these challenges are significant, they should not discourage efforts to genuinely incorporate diverse perspectives. It is crucial to go beyond tokenistic inclusion and ensure that the views of patient partners from varied backgrounds are meaningfully represented [68]. One approach to addressing these challenges is by incorporating a trauma-informed intersectional approach to evaluate patient engagement could offer a deeper appreciation of diversity’s impact [69]. The lack of diversity in patient engagement is recognized [70], with the inclusion of under-represented groups as patient partners being a noted priority [71]. Considering intersectional experiences is critical in the design and evaluation of digital health platforms, which are most effective when they are personalized [27]. Furthermore, factors like race, ethnicity, and socio-economic status can influence digital access and literacy [72]. Investigating inclusive strategies for patients with limited technology access and digital literacy represents an important area for future research. Addressing the “digital divide” by integrating these patients’ perspectives in the early stages of digital health platform development can enhance accessibility [72].

Our scoping review has limitations that warrant acknowledgment. The term “patient partner” was used throughout the article to describe patients who were involved in research teams, regardless of the nature of engagement. We acknowledge that “patient partner” terminology may be ill-suited to describing some of the studies’ involvement with patients. ‘Partnership’ implies a deeper involvement which we did not directly assess. We used this term for concision and with a goal of framing our scoping review within the context of the CIHR SPOR Patient Engagement Framework. Although the Levels of Engagement [50,51] is a common metric for exploring patient engagement depth [73], we opted not to analyze the included studies using this metric due to the dynamic and complex roles of patient partners in research, which make categorization difficult. The original objective was to explore the nature of patient engagement; however, reporting gaps led to subjective interpretations. The interchangeable use of terms like ‘partnership’, ‘involvement’, ‘activation’, and other terminology with patient engagement also introduced challenges in searching and writing this review. We could not assess the use of financial compensation and training opportunities due to a lack of reporting, but these supports should be recognized as a key factor in meaningful and inclusive patient engagement approaches. Additionally, we excluded gray literature due to its lack of rigorous peer review, prioritizing the quality and reliability of our findings. Our criteria specifically targeted peer-reviewed studies that detailed patient engagement in the development or evaluation of digital health platforms for cancer survivors. This focus was crucial for maintaining the integrity and relevance of our results. However, we acknowledge that this approach may have limited the scope of our findings. In addition, it is possible that some studies involved patient partners without explicitly reporting these activities. As with all reviews, our findings are limited to what is described in the published literature and may not fully reflect the extent of patient engagement. We also acknowledge that community health workers, while not consistently described as research partners, represent another way in which lived experience may be embedded in intervention delivery but not captured within our inclusion criteria. Lastly, our search was limited to studies published in English, potentially excluding valuable insights from diverse cultural perspectives on patient engagement, which would require reviewers’ proficiency in multiple languages to fully grasp the associated nuances and terms.

**Fig 3:**
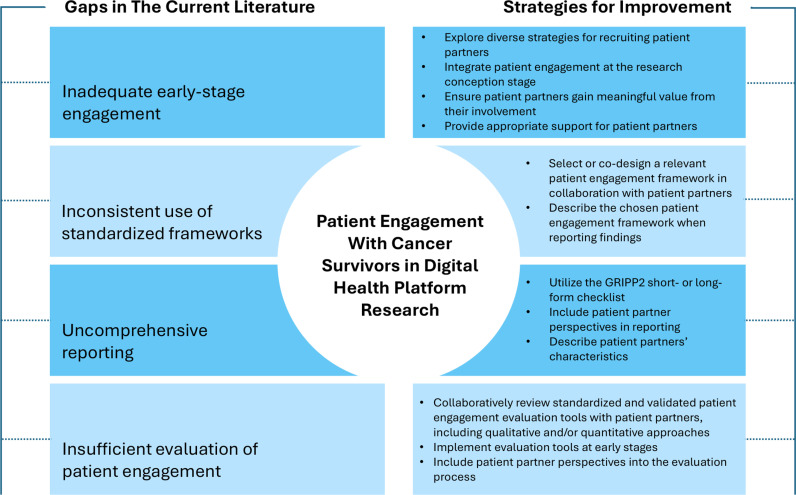
Gaps in patient engagement with cancer survivors in digital health platform research and strategies for improvement.

This scoping review underscores the need for meaningful patient engagement and comprehensive reporting of such efforts in digital health research for cancer survivors. The limited number of studies we identified herein emphasizes that patient engagement remains an undervalued practice, despite a 2017 call to action by the National Cancer Institute (NCI) to prioritize patient and community engagement in cancer research [74]. Among the studies that addressed patient engagement, we identified persistent gaps in implementing strategies and evaluating its impact. These gaps, and strategies to fulfill them, are outlined in **Fig 3**. Addressing these issues is essential for ensuring that digital health research is grounded in the realities and needs of patients. By identifying these barriers, this review aims to inform, as well as foster future studies prioritizing comprehensive, inclusive, and meaningful patient engagement. Furthermore, it provides a foundation for subsequent reviews to assess progress in this critical area, ensuring continued advancements in digital health research for cancer survivors.

## Conclusion

Cancer survivors contribute significantly to digital health research teams with their unique perspectives, suggesting innovative ideas and solutions. However, our review indicates a paucity in description of meaningful patient engagement approaches involving cancer survivors within the digital health platform research sphere. Advancements in the evaluation and reporting of patient engagement are crucial to connect effective strategies with tangible outcomes. Furthermore, addressing digital health research’s unique barriers and meaningfully including diverse patient partners in research teams are essential components to ensure the universality of digital health platforms. Our findings outline strategies, barriers, and unexplored areas in digital health research concerning patient engagement, serving as a guide for research teams planning to involve cancer survivors as partners with lived experience. By fostering future studies and providing a baseline for comparison, this review sets the stage for advancing and assessing progress of patient-oriented approaches in digital health research.

## Supporting information

S1 Checklist Preferred Reporting Items for Systematic Reviews and Meta-Analyses extension for Scoping Reviews (PRISMA-ScR) Checklist. (PDF)

S1 Table Ovid MEDLINE search strategy. (PDF)

S2 Table Detailed examples of patient engagement activities across the research lifecycle in digital health platform studies for lifestyle behavior change among cancer survivors. (PDF)

S1 Protocol Pre-established protocol. (PDF)
