# Supplementary material for: Patient engagement strategies in digital health interventions for cancer survivors: A scoping review
Source: PLOS Digit Health. 2025 May 30;4(5):e0000871. doi: 10.1371/journal.pdig.0000871 (PMC12124549; doi:10.1371/journal.pdig.0000871)
Supplement: S1 Table — (PDF) [file pdig.0000871.s002.pdf]

S1 Table. Ovid MEDLINE search strategy.

Database: Ovid MEDLINE(R) ALL <1946 to May 17, 2023>

Search Strategy:

1 [Patient engagement]

2 exp Patient Participation/ or exp Patient Advocacy/ or exp Patient-Centered Care/ or exp Community Participation/

3 ((patient* or consumer*or stakeholder* or user* or client*) adj2 (perspective* or centered or centred or participa* or collaborat* or partner* or advisor* or advocacy or voice* or unvoiced or involv* or represent* or consult* or contribut* or engage* or activat* or opinion* or dialog* or input* or oriented or co-design* or codesign*)).mp.

4 or/2-3

5 [Digital health]

6 ((health or healthcare or health care or wellness or lifestyle or fitness or exercise* or physical activity or diet* or nutrition* or mental* or stress or behavio?r* or self-help or self control or self management or self care or psychosocial or quality of life or mindfulness or meditation) adj2 (mobile or digital or electronic or web or platform* or software or app* or cell phone* or telemedicine or telehealth or smartphone* or Internet-based or wearable or online or website or iphone*)).mp.

7 (ehealth or e-health or mhealth or m-health).mp.

8 or/6-7

9 [Nutrition, Exercise, Mental health]

10 (wellness or lifestyle or fitness or exercise* or physical activity or diet* or eat* or nutrition* or mental* or stress or behavio?r* or psychosocial or quality of life or mindfulness or meditation).mp.

11 exp Nutrition Therapy/ or exp Diet/ or exp Diet Therapy/ or exp Weight Loss/ or exp Food/ or exp Exercise/ or exp Exercise Therapy/ or exp Physical Fitness/ or exp Resistance Training/ or exp Mental Health/ or exp Mindfulness/ or exp Meditation/ or exp Cognitive Behavioral Therapy/

12 or/10-11

13 [Population]

14 exp Neoplasms/ or exp Carcinoma/

15 (cancer* or carcinoma* or tumo?r* or malignan* or metasta* or neoplasm* or oncolog* leukemia* or lymphoma* or melanoma* or sarcoma*).mp.

16 or/14-15

17 4 and 8 and 12 and 16

18 exp infant/ or exp child/ or exp adolescent/

19 17 not 18

20 limit 19 to english language
