# Supplementary material for: Patient engagement strategies in digital health interventions for cancer survivors: A scoping review
Source: PLOS Digit Health. 2025 May 30;4(5):e0000871. doi: 10.1371/journal.pdig.0000871 (PMC12124549; doi:10.1371/journal.pdig.0000871)
Supplement: S2 Table — (PDF) [file pdig.0000871.s003.pdf]

S2 Table. Detailed examples of patient engagement activities across the research lifecycle in digital health platform studies for lifestyle behavior change among cancer survivors.

Research lifecycle stage	Examples of patient engagement
**Priority setting and planning**	• Involved in persona-development [1]<br>• Engaged in a workshop exploring cancer survivors' needs to set priorities for the intervention [2]
**Development of the research proposal**	• Contributed to a protocol [3]
**Scientific review**	• Reviewed a proposal and proposed edits [3]
**Ethics review**	None
**Oversight of a research project**	• Involved in an advisory and safety committee [4]
**Recruitment of research participants**	• Provided feedback on recruitment materials [3]
**Data collection**	None
**Data analysis and interpretation**	• Reviewed findings from think-aloud interviews [2]
**Knowledge exchange and translation**	• Contributed to effective promotion and publicity strategies for a digital health platform [5]
**Evaluation and quality assurance**	None

1. Adler RF, Morales P, Sotelo J, Magasi S. Developing an mHealth app for empowering cancer survivors with disabilities: Co-design study. JMIR Form Res. 2022;6(7):e37706.
2. Curry J, Roberts H, Smith A, Riley D, Pearson M, Forbes CC. Developing and testing the ExerciseGuide UK website for people with lung cancer: reflections on the added value of patient and public involvement within a doctoral degree. Res Involv Engagem. 2022;8(1):66.
3. Bernard S, Tandon P, Waters A, Selmani S, Wiebe E, Turner J, et al. Preferences, barriers and facilitators regarding virtual pelvic healthcare in individuals with gynaecological cancers: protocol for a patient-oriented, mixed-methods study. BMJ Open. 2023;13(1):e067606.
4. Hanna L, Huggins CE, Furness K, Silvers MA, Savva J, Frawley H, et al. Effect of early and intensive nutrition care, delivered via telephone or mobile application, on quality of life in people with upper gastrointestinal cancer: study protocol of a randomised controlled trial. BMC Cancer. 2018;18(1).
5. Heinen J, Bäuerle A, Schug C, Krakowczyk JB, Strunk SE, Wieser A, et al. Mindfulness and skills-based eHealth intervention to reduce distress in cancer-affected patients in the Reduct trial: Intervention protocol of the make it training optimized. Front Psychiatry. 2022;13:1037158.
