# Supplementary material for: Patient engagement strategies in digital health interventions for cancer survivors: A scoping review
Source: PLOS Digit Health. 2025 May 30;4(5):e0000871. doi: 10.1371/journal.pdig.0000871 (PMC12124549; doi:10.1371/journal.pdig.0000871)
Supplement: S1 Protocol — (PDF) [file pdig.0000871.s004.pdf]

S1 Protocol. Pre-established protocol.

Patient engagement strategies in digital health interventions for cancer survivors: A scoping review protocol

Abstract

Current cancer survivorship care practices lack support for the mental and physical wellbeing of cancer survivors in the areas of nutrition, physical activity, and mental health. Digital health platforms, including mobile apps and websites, can provide these forms of healthcare to cancer survivors and reduce the need for direct support from healthcare providers. For digital health to be effective, the interventions they deliver should align with the needs of their intended patient population. Engaging patient partners in the design and research of digital health platforms can improve the suitability of interventions to patients and potentially improve their cancer survivorship care.

The objective of this scoping review is to explore the engagement of patient partners in research involving digital health platforms and cancer survivors. The specific objectives are to 1) identify strategies that have been used to engage patients in research; 2) identify characteristics of successful patient engagement; 3) explore challenges and gaps in research involving patient partners.

The scoping review will follow the Joanna Briggs Institute methodological framework. Medline, EMBASE, and Scopus will be searched for topic-related keywords. Two independent reviewers will screen the identified articles using Covidence and conflicts will be solved by consensus or a

third reviewer. Data extraction will be conducted with the same process, using a pre-established, piloted, extraction chart.

Introduction

Over the last thirty years, cancer survival rates have greatly improved (1–3). However, cancer survivors often experience poorer mental and physical health than those without chronic diseases (4). Cardiovascular problems are common among them and a major cause of death, highlighting the need for effective interventions to improve their well-being.

Encouraging lifestyle changes that involve exercise, nutrition, and mindfulness can help lower cardiovascular disease risks and enhance the quality of life for cancer survivors, potentially increasing their chances of survival (5–7). Cancer survivors often do not receive these interventions due to their costs and the availability of healthcare professionals, necessitating programs that provide comprehensive care to many while reducing the strain on healthcare systems.

Digital health platforms offer a potential solution to deliver healthcare widely and reduce the burden on healthcare systems. Digital interventions have shown success in promoting physical activity among cancer survivors (8). However, more research is needed to assess the effectiveness of digital approaches for nutrition and mental health. Customizing these interventions to fit patient needs is crucial for success (9). Engaging patient partners during the development and testing of digital health interventions can align them better with patient needs, improving the delivery of healthcare.

Many health research institutions worldwide promote patient involvement in research teams, using different frameworks. The article will use the term ‘patient engagement’ as defined by the Canadian Institute of Health Research (CIHR) Strategy for Patient-Oriented Research (SPOR) (10). Patient engagement is becoming more commonly applied to research but reporting and evaluation of its implementation remain limited in the literature (11).

The aim of this scoping review is to explore the state of patient engagement in research involving self-management digital health platforms for cancer survivors. Our specific objectives are to 1) identify strategies that have been used to engage patients in research; 2) identify characteristics of successful patient engagement; 3) explore challenges and gaps in research involving patient partners.

Review question

The review centers around the following question: Has patient engagement been incorporated into research on digital healthcare interventions for managing nutrition, physical activity, and mental health in cancer survivors? A subsequent set of questions were answered by the literature and guided the extraction process: (1) What strategies have been used to engage patients with cancer, or their caregivers, as patient partners in research involving digital healthcare? (2) Have these strategies been effective? If so, what are the characteristics of successful patient engagement in digital health interventions? (3) What are some challenges in engaging cancer survivors or their caregivers, as patient partners in digital health research? What areas of patient engagement research in digital health interventions are lacking?

Keywords

Digital health; patient engagement; cancer survivor.

Eligibility criteria

Participants

Eligible studies will include survivors of cancer. The classification of cancer survivorship in the eligible studies will be broad, encompassing patients at all stages of disease after their initial diagnosis, according to the National Cancer Institution's definition (12). Only adult patients, or their caregivers, as direct partners will be studied. Caretakers of young individuals or parent engagement will not be included; thus, the search will exclude research involving infants,

children, or adolescence. Studies with adult and pediatric cancer survivors will be considered only if a separate description of patient engagement strategies for each population is provided.

Concept

Research that is considered to have used patient engagement strategies must have actively engaged patients or their caregivers at any step of the research process, from digital platform development to validation. The inclusion of patient partners must be explicitly outlined. To align with the CIHR definition of patient engagement, patient partners can be patients or informal caregivers who contributed to the research or development process but have not been a part of the data set. This definition also encompasses other frameworks for patient engagement.

Context

The studies will use digital platforms to deliver healthcare interventions in the following areas, independently or in combination: nutrition, physical activity, and mental health. The digital intervention may use web-based platforms or digital apps. Wearable technology and health record tools, as well as text message, will only be considered if they are used in conjunction with a digital platform to deliver one of the specified areas of health.

Types of sources

The sources that will be included in this scoping review are clinical trials, observational studies, and trial protocols that test digital health interventions. Qualitative studies or mixed-methods studies that assess patient engagement strategies in digital health interventions will also be considered for inclusion. Thesis, case reports, case series, narrative reviews, systematic reviews, meta-analysis, conference abstracts, and non-peer-reviewed articles will not be included.

Methods

The scoping review will follow the methodological framework outlined by the Joanna Briggs Institute (JBI) (13), which was refined from the original framework developed by Arskey and

O'Malley (14). Reporting of the review will align with the Preferred Reporting Items for Systematic reviews and Meta-Analyses extension for Scoping Reviews (PRISMA-ScR) checklist (15).

Search strategy

First, a search will be conducted on Ovid MEDLINE to identify relevant articles and derive index terms. Secondly, a refined search strategy will be formed, first for Ovid MEDLINE, and adapted for Ovid EMBASE and Scopus. The adapted search strategy for each database will be made through modification of the established keywords and index terms. Search terms related to the following concepts: *patient engagement*, *digital health*, *lifestyle intervention*, and *patients with cancer* will form the basis of the search strategy. The search will be conducted on May 23, 2023.

Study/Source of Evidence selection

The articles found through the search will be extracted and uploaded into Covidence, which will be used to guide the source selection. Duplicate articles will be automatically removed before screening. Two or more reviewers will independently screen the titles and abstracts followed by the full texts of the eligible articles. Each reviewer will independently review the full texts in reference to the eligibility criteria. Disagreements between reviewers will be resolved by consensus. The process will be reported in a flow diagram as outlined by the PRISMA-ScR statement and a description of the process will be provided (15).

Data Extraction

A data extraction form will be adapted from the data extraction template created by the Joanna Briggs Institute, which will guide data extraction from each article by two independent reviewers. To ensure that the extraction form includes all necessary information, the form will undergo a pilot test with two articles before continuing the data extraction step. Any other modifications will be documented. The data extracted from the articles will include: citation details, title, country, type of study, condition of participants, age of participants, number of participants, type of digital intervention, area(s) of health intervention used, patient

engagement strategies used, CIHR SPOR research lifecycle involved (16,17), and reporting of patient engagement against the Guidance for Reporting Involvement of Patients and the Public 2 (GRIPP2) checklist (18).

Data Analysis and Presentation

A flow diagram will be used to depict the article selection process. The diagram will specify the number of articles assessed and the process used at each stage. The reasons for exclusion and number of excluded articles will also be shown. A summary of study characteristics will be presented in table format and described–some characteristics will be presented in visual format. The CIHR SPOR research lifecycle stages (16,17), where patient partners are involved, will be depicted in a table. Another table will list the patient engagement framework used, terms used to define patient partners, the descriptions of the patient partners, and the number of patient partners. The outcomes of the study, barriers for patient engagement, and reporting of patient engagement will be described. The reporting of patient engagement will also be presented in a table with the items of the GRIPP2 short form (18).

Acknowledgements

Funding

This work was supported by Alberta Innovates (grant reference: RES0061458) and by the Government of Canada through the Canadian Institutes of Health Research (grant reference: 202209PJT). This research was also undertaken, in part, thanks to funding from the Canada Research Chairs Program to CMP. MR is supported by a Summer Studentship from the Cancer Research Institute of Northern Alberta and the Alberta Cancer Foundation. CEO is supported by the Mitacs Accelerate program (IT29265), a collaborative funding program with My Viva Inc.

Conflicts of interest

The authors declare no conflicts of interest associated with this study.

References

1. Brenner DR, Poirier A, Woods RR, Ellison LF, Billette JM, Demers AA, et al. Projected estimates of cancer in Canada in 2022. CMAJ. 2022;194(17):E601–7.

2. Ellison LF. The cancer survival index: Measuring progress in cancer survival to help evaluate cancer control efforts in Canada. Health Rep. 2021;32(9):14–26.

3. Santucci C, Carioli G, Bertuccio P, Malvezzi M, Pastorino U, Boffetta P, et al. Progress in cancer mortality, incidence, and survival: a global overview: A global overview. Eur J Cancer Prev. 2020;29(5):367–81.

4. Stein KD, Syrjala KL, Andrykowski MA. Physical and psychological long-term and late effects of cancer. Cancer. 2008;112(S11):2577–92.

5. Kim KH, Choi S, Kim K, Chang J, Kim SM, Kim SR, et al. Association between physical activity and subsequent cardiovascular disease among 5-year breast cancer survivors. Breast Cancer Res Treat. 2021;188(1):203–14.

6. Ligibel JA, Bohlke K, May AM, Clinton SK, Demark-Wahnefried W, Gilchrist SC, et al. Exercise, diet, and weight management during cancer treatment: ASCO guideline. J Clin Oncol. 2022;40(22):2491–507.

7. Wayne SJ, Baumgartner K, Baumgartner RN, Bernstein L, Bowen DJ, Ballard-Barbash R. Diet quality is directly associated with quality of life in breast cancer survivors. Breast Cancer Res Treat. 2006;96(3):227–32.

8. Haberlin C, O'Dwyer T, Mockler D, Moran J, O'Donnell DM, Broderick J. The use of eHealth to promote physical activity in cancer survivors: a systematic review. Support Care Cancer. 2018;26(10):3323–36.

9. Roberts AL, Fisher A, Smith L, Heinrich M, Potts HWW. Digital health behaviour change interventions targeting physical activity and diet in cancer survivors: a systematic review and meta-analysis. J Cancer Surviv. 2017;11(6):704–19.

10. Canadian Institutes of Health Research. Strategy for Patient-Oriented Research: Patient Engagement Framework [Internet]. 2014 [cited 2023 Dec 15]. Available from: https://cihr-irsc.gc.ca/e/48413.html

11. Weschke S, Franzen DL, Sierawska AK, Bonde LS, Strech D, Schorr SG. Reporting of patient involvement: a mixed-methods analysis of current practice in health research publications using a targeted search strategy. BMJ Open. 2023;13(1):e064170.

12. National Cancer Institute. Cancer survivorship [Internet]. 2014 [cited 2023 Dec 15]. Available from: https://www.cancer.gov/about-cancer/coping/survivorship

13. Peters MDJ, Godfrey CM, Khalil H, McInerney P, Parker D, Soares CB. Guidance for conducting systematic scoping reviews. Int J Evid Based Healthc. 2015;13(3):141–6.

14. Arksey H, O'Malley L. Scoping studies: towards a methodological framework. Int J Soc Res Methodol. 2005;8(1):19–32.

15. Tricco AC, Lillie E, Zarin W, O'Brien KK, Colquhoun H, Levac D, et al. PRISMA extension for scoping reviews (PRISMA-ScR): Checklist and explanation. Ann Intern Med. 2018;169(7):467–73.

16. Canadian Institutes of Health Research. Ethics Guidance for Developing Partnerships with Patients and Researchers [Internet]. 2020 [cited 2023 Dec 15]. Available from: https://cihr-irsc.gc.ca/e/51910.html

17. Anderson JA. Research Ethics Broadly Writ: Beyond REB Review. Health Law Rev. 2011;19.

18. Staniszewska S, Brett J, Simera I, Seers K, Mockford C, Goodlad S, et al. GRIPP2 reporting checklists: tools to improve reporting of patient and public involvement in research. BMJ. 2017;358:j3453.
